# Being differently abled: Disability through the lens of hierarchy of binaries and *Bitso-lebe-ke Seromo*

**DOI:** 10.4102/ajod.v9i0.643

**Published:** 2020-02-25

**Authors:** Paul L. Leshota, Maximus M. Sefotho

**Affiliations:** 1 Department of Theology and Religious Studies, National University of Lesotho, Maseru, Lesotho; 2 Department of Educational Psychology, University of Johannesburg, Johannesburg, South Africa

**Keywords:** being differently abled, disability, hierarchy of binaries, *Bitso-lebe-ke seromo*, naming, identity formation

## Abstract

**Background:**

Despite its acceptability, the term disability has not been able to shirk the sense of incompleteness, lack, deprivation and incapacitation embodied in the prefix ‘dis-’. The current wave of anti-discrimination on disability issues, calls for constant re-examination of the language and the appellations we use in respect of people with disabilities.

**Objectives:**

The aim of this study is to subject the term disability to some relevancy litmus test with a view to prevent it from acquiring Lyotard’s ‘grand narrative’ and to propose and argue for the term ‘differently abled’ because of its transformative and anti-discriminatory slant.

**Method:**

The study took the form of a literature review using the optic of Derrida’s hierarchy of binaries and the Sesotho proverb, ‘*Bitso-lebe-ke seromo*’, (A bad name is ominous) to explore the connotations of the term disability as a disenfranchising social construct.

**Results:**

Read through the lens of Derrida’s idea of difference, disability as a concept has no inherent meaning and its meaning derives from its being differentiated from other concepts. Viewed through the lens of *Bitso-lebe-ke seromo* and read in the context of its deep symbolical significance, the term disability holds immense spiritual power.

**Conclusion:**

The study concludes that the term disability or disabled is exclusionary, stigmatizing, and anti-transformational. As such it embodies imperfection, incapacitation and inferiority. Not only is it ominous, it places upon people with disability the perpetual mark of unattractiveness. Against this background the term differently abled seems to convey more empowering overtones than the term disability.

## Introduction

A great deal has been written about the vital role that language plays in identity formation within the framework of culture (Corker 1999; Galvin 2003; Sheer & Groce 1988; Wittgenstein 1994; Wright 1960). Language has been seen as more than just an instrument of communication to convey ideas between people. Language, in some cultures, has served to shape people’s behaviours (Wittgenstein 1994), while in other cultures it has been perceived as having a causal effect, that is, to bring about what it signifies. Naming as an aspect of language has also been a subject of intense debate, especially in the creation of identity (Mulhausler & Harre 1990; Swain & Cameron 1999; Woodward 1997). The shared belief of the above voices is that the process of naming creates a subject whose sense of self is connected with the society’s definition (Galvin 2003:152). In this way, individuals are recruited into identifying with labels and identities created not by them but by society.

To the question, ‘What’s in the name?’ asked by Galvin (2003:153), the answer might be, ‘there is power in a name’. Naming or labelling, as Lynch (2016:208) observed, is more than just an identity marker. It is a political act with the power to include and exclude (Barnes 1992:8). By means of naming, which is a linguistic device, one is subjected to someone else by control and dependence. The force of that power and control is given meaning by the culture within which that naming, as a linguistic device, takes place (Leshota 2011:119). Because naming is not only a value-free exercise but also a connoting act which shapes perceptions, individuals and groups of people have had to bear with appellations that shaped their perceptions about themselves and others in negative and disempowering ways (Lynch 2016:208). In the process, negative and enduring stereotypes as well as undesirable identities were created about such people.

It is in this context that terms such as ‘cripple’, ‘invalid’, ‘spastic’ and ‘freaks’ were used with respect to people with disabilities (PWDs). While these appellations may have been appropriate for their time, the treatment of the people so named was a reflection of what society thought about them. They were the property of the political hegemony that could be displayed for entertainment purposes because of what was perceived to be their unusual physical appearance. Their association with such stigmatising images ascribed some characteristics that functioned to delegitimise them (Lynch 2016:208). With time, such appellations were challenged as inappropriate because they were found to conjure intensely negative images and representations (Galvin 2003:157). To date, the term PWDs has been accepted within movements of people with disabilities across the globe as the most fitting and appropriate referent. Our contention is that with the passage of time, everything needs to be subjected to some relevancy litmus test to save it from acquiring the status of Lyotard’s ‘grand narrative’. We, therefore, are spurred by what Slee and Allan (2001:180–181) called ‘scholarly and cultural vigilantism’ in our effort to look for how things could be improved particularly because disability, as Obosi (2010:12) observed, is an area where language is subject to debate and change.

This article uses Derrida’s philosophical notion of deconstruction under its aspect of hierarchy of binaries, with its implied centres, to tease out the usage of the term ‘disability’ and look at how it might uphold meanings it intends to flush out. It further uses the optic of the Sesotho proverb, *Bitso-lebe-ke seromo* – read within its own Sesotho world view – to explore the cultural connotations of the term ‘disability’ as a disenfranchising social construct and encourage adoption of the phrase differently abled as an alternative to disability. The deployment of the above can provide valuable lenses in interrogating the identification of people through their disability, as setting them apart, distinguishing and separating them, thus removing them from the centre, which has a tendency to exclude, marginalise, vilify and disempower (Powell 1997:21).

## History of the concept of disability and the creation of disabled identities

Perceptions towards those that are different in general and PWD in particular have varied from time to time and from one culture to another (Munyi 2012). Both culture and history connived, through language, practice and ritual, to construct perceptions and therefore meanings around bodies that were considered different and abnormal. Society, with its worldview and not biology, therefore, determined the acceptability of bodies and what meanings they should inhere. Wendell (1996) opined that disability is socially constructed in varied ways ranging from social conditions, physical functioning, to subtle cultural factors that have, for years, determined what qualified as normal and therefore acceptable and what qualified as abnormal and was therefore excluded.

Throughout the years, attitudes fuelled by perceptions, reinforced through language and practice, have never remained static. The Greco-Roman culture has consistently idealised bodily perfection. Garland (1995), Stiker (1999) and later Davies (2000) and Rose (2013) have focussed on the development of the concept of disability in history, particularly its derivation from Greco-Roman culture. Although there are varied nuances in their conclusions, they seem to agree that disability as a social construct deriving from the biological category of impairment was present in the Greek and Roman cultures. Names and appellations attributed to people, who were considered physically unable to meet the standards as determined by society, were not lacking. Words such as *monstrum, mutus, debilitas, infirmus, invalidus* and *deformis* are quite common in Roman literature to represent PWDs. While *monstrum* has nuances of a subhuman with the possibility of abandonment in the case of a child, other words imply weakness, inability, feebleness, ugliness, deformity and debility.

In ancient Greek, the term αδυνατος did mean something akin to disabled. To such people were accorded pity and charity. They were further exempted from military service and politics. The Greeks and the Romans placed great value on competition, war and sport, and their bodies had to be such that they could participate successfully in all these activities (Garland 1995:14). Physical and intellectual fitness were esteemed features in both world views, as they ensured triumph and conquest in any form of competition (Barnes 1997:13).

Similarly, the ancient Israelites, like the Greeks and the Romans, and other societies attributed meanings to bodies and the criteria under which they would be judged as normal, natural, perfect and whole. They espoused a regulatory body, which is a body against which all other bodies were measured. According to Douglas (1966:115), such a body served as a microcosm of a social body. It therefore exposed the society’s deepest convictions and values on everything else including the body.

All of this depended entirely on the societal reckoning of what should constitute an acceptable body. According to the ancient Israelites, the body was perfect and therefore clean, or it was imperfect and therefore unclean if it did or did not meet certain physical or aesthetic conditions. A perfect body had to meet the criteria of wholeness, maleness and godlike features. These features defined membership and belonging within the hierarchical structure (Malina 1981:122).

The Jewish tradition, alive in the Hebrew Bible and mentality – on the whole – attributed impairment to divine ordination resulting from sin of people or their parents on the basis of the principle of corporate personality. Such persons whose identity was associated with blindness, lameness, mutilated face, excessive limbs, injured hand, hunched back, dwarfism, itching disease, scabs and crushed testicles were considered defective. The above conditions constituted incompleteness and impurity, which were seen as an affront to God who was holy and without blemish (Lv 21:8). The Torah forbids people to serve God under the condition of *tameh* (pollution). There are, however, some Hebrew Bible texts that portray disability in a positive light. Leviticus 19:14 has the tone of an anti-discrimination law, protecting, as it were, the deaf and the blind from harassment.

With Jesus’ coming on the stage, people with all forms of maladies and disabilities became the focus of his ministry. Jesus’ healings were occasions for not only physical healing but also an opportunity for the sequestered to be reintegrated into society. While the names may have remained the same, the attitude towards people with bodies that did not meet the standards of a regulatory body was greatly challenged by Jesus’ disruptive position. As Stiker (1999) observed:

[*I*]n going out to those who were under the interdiction (lepers, the blind, prostitutes, etc) or in letting them come to him, he was performing less a social act than an act to deconstruct the religious mentality. (p. 33)

Under the new dispensation, it is not anymore about ritual purity but about a pure heart.

Throughout the history of the Western Christian Tradition, disability and disabled people have continued ‘to surface as that which must be assimilated or made to disappear’ (Stiker 1999:xi). The individualisation and medicalisation of the body and the mind led to the further exclusion of PWDs and their confinement into institutions. The eugenic ideals that led to the systematic extermination of PWDs in the Nazi camps, under the pretexts of achieving a ‘Utopian society’, came as no surprise. This negative perception notwithstanding, an upsurge of Christian charities continued to exist alongside the former and influenced society’s perception of disability in different ways. It was in the 19th century that different coinages and appellations around the realities of disability were designed in keeping with the social and human rights trends of the time. While these coinages may have been accepted in certain sections of society, the debates on how best to arrive at appellations that are contextually germane while being globally appealing are raging on. Taking cue from these debates, we are adding our voice to the debate.

## Disability under the lens of Derrida’s deconstructive hierarchy of binaries

One of Derrida’s contributions to the post-modern and post-structuralist paradigms was his coining of the term ‘deconstruction’. While post-structuralism posits that meanings carried by words are not fixed but always temporary (Burr 2003:53), and that such meanings are dependent on words as used in the context of time and place, deconstruction, from Derrida’s perspective, is described as a ‘way of reading that concerns itself with decentering – with unmasking the problematic nature of all centres’ (Powell 1997:21). This stance was a reaction to the influence of Western metaphysics, which saw the world as founded on a centre. That centre was viewed as an ideal form and a fixed point around which meaning is generated.

Disability as a concept has no inherent meaning. Its intended meaning derives from derive from its comparison with and differentiation from other concepts. Because language, working through concepts, is founded on relation, the meaning of concepts is dependent on their being elements in a system of differences (Powell 1997:21). Disability’s meaning therefore depends on its relation to its opposite in the system of differences. It is the opposite of abled or able-bodied. Not only is disability the opposite of able-bodied, but the latter is more privileged than the former. According to Redman (2000:12), the notion of able-bodied is ‘constantly haunted by the liminal presence of the disabled others against which it defines itself and into which it continually threatens to collapse’. The taken-for-granted assumptions about disability’s meanings collapse in the face of their refusal to remain linguistically stable (Galvin 2003). Derrida’s notion of deconstruction allows for the questioning of these taken-for-granted assumptions and renders their subversion possible. History bears testimony to the fact that the disabled body has, throughout the years, been subjected to a variety of socially generated interpretations. Almost all of these interpretations and meanings were founded on the hierarchy of binaries with their implied power relations. The meanings around these binary opposites should be subjected to scrutiny, and deconstruction affords us the scope and the means.

In this relationship of abled versus disabled, in the system of binaries, not only are the terms opposed, but one, abled in our case, is always privileged over disabled, evoking as it were, relation of dominance. As such, it occupies the centre and thereby generates meaning that marginalises disabled or any category that falls outside the purview of the centre. Not only does able-bodiedness occupy the centre, it also functions as a fixed regulator and a measure of all the other bodies (Leshota 2011:54–55). Disabled bodies have, throughout history, been found wanting. They were identified as the embodiment of sin and sinfulness, as the incarnation of tragedy by the moral and the medical models, respectively. The notion of disabled bodies makes sense, therefore, within the ‘othering’ discourse that sets up a division between normal and abnormal. Within such a discourse, able-bodiedness becomes the normal, the perfect, the desired and that which must be maintained at all costs. Disability, on the contrary, represents the abnormal, imperfect, in its physical and moral sense, which is sustained by the desire to flee from itself towards the norm and the perfect. Until such a desire is fulfilled, disability cannot rest, and it will forever remain the ‘other’ that must disappear (Stiker 1999:xii).

Not only does the term ‘disability’ carry this abnormalisation and ‘othering’ connotations, it further imposes on the named demeaning and stigmatising associations. Within religious contexts, they constitute, for the most part, a group that is seen as sinful and unwhole, and therefore in need of healing and redemption (Leshota 2011:144). One person with disability, I had met in one of my errands, had these words to share: ‘I have since stopped going to church because I still feel treated like an outsider’. This experience resonates with similar other experiences, where although other PWDs have not left the church, they still feel the church could do better in its treatment of PWDs (Njoroge 2001:7). Within developmental contexts, in spite of the many commendable efforts made, in the form of policies, conventions, laws and commitments, employment opportunities for PWDs in developing countries are often almost non-existent.

Consequently, many PWDs have to beg for a living, whereas, in actual fact, ‘employment is the only way out of lifelong exclusion’ (Okola 2011:147). Within educational contexts, in spite of the UNESCO EFA Flagship and the major strides made in awareness raising which led to change of attitude, pedagogical processes and built environment are still not disability-friendly. This has resulted in the fact that very few learners with disabilities in Africa go past primary school (Miles & Ahuja 2007). With no education and skills to negotiate the competitive economic environment, PWDs are not empowered to fight poverty. Within the health systems, most PWDs in developing countries still have no access to medical and rehabilitation services (WHO 2007). In Lesotho, and possibly in many other resource-constrained countries, where even access to bare nursing services takes months to happen, provision of sign language interpreters for patients with hearing and speech impairments or Braille facilities for patients with visual impairments would be a luxury.

Access to justice still has a long way to go in terms of reasonably accommodating PWDs into the justice system (Larson 2014). While there is some encouraging progress in some countries, there are equally varied challenges for some countries to bring to fruition commitments made with respect to access to justice for PWDs. Challenges range from training of personnel, policies and laws, attitudes, infrastructure to legal systems themselves. In Lesotho, for example, the justice sector is still steeped in the rehabilitation and deficit model of disability, which views PWD as lacking in something that must be restored before they can be resettled into society (Constitution of Lesotho 1993). Both in principle and practice, PWDs have been declared incapable of participating in issues of justice that concern them. Courts still rely heavily on testimony by eyewitnesses and so people with visual impairment are as a result sidelined by the justice system.

While it may not be inferred that negative treatment results directly from the use of the term ‘disability’ on PWDs, as in the cause–effect relationship, it cannot be at the same time ignored that the term ‘disability’ evokes very negative associations that have had far-reaching implications for its referent, persons with disabilities and their welfare. The long history of marginalisation for PWDs has only proven to us that new models and attitudes take years to develop distinct and liberating contours (Bosch 1991). In spite of the many years since Jesus’ rapture of the preceding mentality against PWDs and the church’s compassionate attitude against PWDs throughout the years, the church is yet to make a break from the old mentality and embrace Jesus’ liberating praxis. Society, too, has not fared any better. There are as many good stories as there are sad stories to tell with respect to PWDs (Retief & Letšosa 2018). Disability’s location within the framework of the hierarchy of binary opposites renders it suspect and therefore wanting in terms of fairly representing positive and constructive meanings for PWDs. As Dunne (2009:48) suggested, it upholds meanings it intends to flush out. On the basis of the above consideration, we strongly argue for its replacement.

## Disability through the optic of *Bitso-lebe-ke seromo*

Although quite a very complex category, scholars have agreed that culture is a collective experience of people who happen to inhabit the same world view (Lartey 2003:31). It expresses itself through language, ritual and practice. Within this experience are embodied wisdom, values, beliefs and practices of how people who inhabit and share the same worldview ought to live. It functions to provide order and give meaning to people’s behaviour and interactions. Although something of its past always remains, culture will forever remain dynamic, adaptable and therefore subject to reinterpretation.

Disability is a culturally mediated category. Its meanings and connotations are determined by the norms of the culture within which it exists. Culture shapes us into who we are, and we, in turn, construct culture. Language plays an important role in the understanding of ourselves as a culture. Language and culture are inseparable. In fact as Mphande (2006:105) puts it, ‘Language is part of culture’. Lotman (1978:211) concurs and further states, ‘No language can exist unless it is steeped in the context of culture; and no culture can exist which does not have at its centre, the structure of natural language’. Agyekum (2006:211) called it an exit valve through which people’s beliefs and thoughts, and cognition and experiences are articulated. It serves not only for communication and sharing of ideas; it goes further to shape as well as to guide the experiences of those who use it.

One linguistic device that has been in use among African communities, who relied mostly on oral culture, is the proverb. A proverb is not simply a tool to advance and enhance good communication; it is also regarded as a deep symbol within culture that reveals the world view of the people. One proverb, in popular use among the Basotho of Lesotho, is *Lebitso lebe-ke seromo*. Literally translated: ‘A bad name is ominous’. What this proverb reveals about the African world view in general and that of the Basotho in particular is that a name is more than just a social identifier. It serves to represent reality and through it reality is known. As a sign, it points towards the individual who is signified by the name within the linguistic structures and patterns provided by culture. Over and above its identification and differentiation roles, the name also holds an immense spiritual power to ‘reflect and indexicalise the lives and behaviour of people either positively or negatively’ (Agyekum 2006:231). It carries the very being of a person. In the context of what students of cultural anthropology and sociology of religion call presentational symbolism, a name has an inherent ability not only to point towards what the name signifies but also to generate or bring about what the name signifies (Hubbeling 2009). Because a name carries such an immense power, and for that matter the soul of a person, it could determine a person’s destiny. A good name spelt a bright future and in the same vein a bad name, except if it is given for preventative^1^ or survival reasons (Agyekum 2006:231), was a bad omen to the child. It is in this sense that a name could be considered to be ominous. It was within this context that sorceries or witchcraft practices could be effected on people by the mere use of a name, without the owner’s presence.

The term *bokooa* (disability) and its cognate *sekooa* (person with disability) is, in Sesotho lexicons, defined in terms of *boholofali* (impairment), *bokulane* (illness) and *boqhoala* (permanent incapacitation) (Pitso 1997:56). The word has connotations of paralysis and complete dependence. *Bokooa* as a term seems to predate the era of the disability movement. Its use as a generic term for all forms of disabilities in Sesotho is quite recent. It is an attempt to match Western categorisation, which creates, through surveys, projects, public systems and policies, the disabled as a social category (Ingstad & Whyte 1995). Historically, the Basotho had specific terms and conceptual categories for persons who had this or the other perceived difficulty or problem. Even people who, although normal by today’s disability standards, were not responsive to society’s usual expectations were regarded as abnormal (Guma 1971:53). These were generally people with mental, moral and physical defects (Leshota 2011:98).

*Bokooa* has all the signs of something undesirable, dreaded and wished away in society. For example, a Sesotho proverb, *Monna o pata sehlotsa* (literally a man hides his limp), is suggestive of the fact that a limp (physical impairment) is a weakness that has to be hidden. It ought to be hidden because it reveals physical unwholeness and deformity, which were dreaded and abhorred in society. If disability is so much disliked and dreaded in society, no member would wish it upon any one member of the family. It is becoming common these days to hear people using *bokooa* to refer to irrationality, incongruity, senselessness and absurdity. People would refer to someone’s argument as having *bokooa* to mean it is absurd.

For one, therefore, to want to give a name or keep on calling a name that is so unattractive and which represents something that society so dreads is calling upon oneself something one would not be able to live with. To keep on calling such a name is - within the context of Sesotho world view - an invitation of a misfortune or omen. *Bokooa* is considered a bad name which carries negative and derogatory overtones used to demean and undermine other people.

## Arguing for ‘differently abled’ as an alternative to disability

What we have been able to discover through the analysis above is that a body is a social construct and that its understanding depends on socially generated interpretations. Society through language and its use continues to construct people, especially those perceived to have a lack or a disability. What has emerged from the discussion above is that the word disability is a negation of ability. As Jones (1996:347) opined, it is construed in an ‘oppositional relationship to ability’. It depends on its opposite for its existence and to fully represent what it signifies.

The process of normalisation of or regularising the body, which has been orchestrated through and by means of binary opposites, is fraught with political ramifications. It is founded on the binaries of the regulariser versus regularised; the normer versus the normed upon; the namer versus the named; the abled versus the disabled, with power valences skewed in favour of the first members of these binaries. The able-bodied are the regulariser, the normer and the namer. The disabled are the regularised, the normed and the named. As such they are marginalised, objectified and subjected to someone by control and dependence (Galvin 2003:150). By participating in the process of naming the disabled, we become accomplices in sustaining the politics that ‘set up a symbolic frontier between the aberrant and the normal’ (Galvin 2003:154). Within the framework of binary opposites, the language of disability is not only subjugating towards people considered disabled by society it is also a disenfranchising social construct.

The ideas of Derrida, in particular, the notion of deconstruction, the centre and the binary opposites, have allowed us to expose the often glossed over power dynamics inherent in the able-bodied versus disable-bodied binary. The use of disability as a term evokes strong feelings of inadequacy, deficit, dependency, abnormality, objectification and waiting to be rescued. It further ‘produces certain consequential effects upon the feelings, thoughts or actions’ of the affected individuals and the wider society (Austin 1962:101).

In light of the lens of *Bitso-lebe-ke seromo*, which make sense within the context of the African (Basotho) worldview, *bokooa* is a bad name; it is a negative language that invites, through the spiritual powers inherent in it, misfortune. On the basis of the fact that language can enliven or kill, naming as an aspect of language has the capacity to give life and to kill. By means of a name and given that a name, in an African world view, holds powers to reflect, indexicalise and symbolise both representationally and presentationally on the basis of the belief that it (name) carries the soul of a person, giving an ominous name is tantamount to condemning a person to a life sentence.

We argue that the term ‘disabled’, on the basis of the above reflection, is exclusionary, stigmatising, demeaning, marginalising, disenfranchising, counter-developmental and anti-transformational. It further embodies inferiority, abnormality, imperfection, incapacitation and dependence. Not only is it ominous, it also places upon PWD a perpetual mark of unattractiveness, which nobody would wish upon himself or herself. Through such an appellation, PWDs are reminded of the feature of ‘not-having’ or incompleteness expressed in the prefix ‘dis-’, which stands for deprivation or in other cases the contrary or the opposite. The prefix therefore deprives PWD of the feeling of being able, capable, capacitated, competent and empowered. Although it is still the accepted term in general use, it seems to be overtaken by current changes that call for inclusion and transformation. A transformative difference is promoted through embracing the phrase differently abled, which was first proposed (in the 1980s) as an alternative to disabled, handicapped and other demeaning terms traditionally used on the grounds that it gave a more positive message and so avoided discrimination towards PWDs.

While the term ‘disability’ may have been discounted on the basis of its negative associations, we are yet to argue for the adoption of the term ‘differently abled’. The long history of disenfranchisement and negative treatment towards PWDs has eventually seen efforts being made not only to demand better treatment by PWDs but also to shirk the labels that are detrimental to the image and dignity of PWD (Galvin 2003:7). It was in this context that the term ‘differently abled’ was coined in the United States in the early 1980s. It soon started to gain traction in society and in church. Kabue (2016:213) observed that the term ‘differently abled’ was embraced and used within the circles of the World Council of Churches until it was supplanted by the terms ‘persons with disabilities’ and ‘disabled persons’. The term, though, has not completely died out. It still raises its head in protest (Obosi 2010). In light of the ever-changing interplay between language, under the aspect of naming, and relationships, the term should remain the candidate for the category of disability.

Our arguments in favour of differently abled derive from anthropological, linguistic and legal considerations. Firstly, the adoption of the term ‘differently abled’ is founded on the conviction that PWDs are fully human, endowed with personal dignity and therefore deserving of the same respectful treatment that is accorded to every human. The term ‘differently abled’ seems to shirk the burden of deficit that is carried by the prefix dis-, representing, as it were, a lack or a deficiency. It further has proclivity for empowerment and human transformation. Secondly, naming is one way in which someone is assigned a set of characteristics, which, according to Lynch (2016:208), legitimised or delegitimised such a person. A name is imbued with meaning that derives from culture. As such, it influences attitudes and thoughts that people, within that culture, have about people who are named. It is in this sense that Obosi (2010:6) proposed a disability-friendly language both in intention and execution. This simply points to the reality of diversity, which is a truly human feature. While we all share in the same humanity, we do so, each one of us, in different and unique ways. The term further emphasises abilities that as humans we all enjoy, in spite of the differences and limitations that each one of us has (Woodhams & Danieli 2000:405). Differently abled promote abilities that may be different from those deemed normal, and are celebrated because they make life liveable. Difference in this sense is construed to imply diversity, not inability or lack of abilities. Dei (2004:345) warned that: ‘difference cannot be accentuated for its own sake’. Humanity should, on the contrary, see beyond the myriad of differences, to possibilities of collective strength for more sustainable livelihoods. With a shift from disability to differently abled, a shift from binary and dichotomous pairs – which survive on unequal and oppositional relationship – is achieved. With the adoption of the term ‘differently abled’, the implied comparison between the able and the disabled is highly reduced. Lastly, the fact that differently abled is founded on the dignity inherent in humanity leads into the human rights discourse, where, according to Obosi (2010:6), getting the language right to match the human dignity in PWDs is non-negotiable. In light of the above arguments, we propose the re-consideration of the term ‘differently abled’ to replace disability.

## Conclusion

No word has an inherent meaning. Every word derives its meaning from the context within which it is used. The multiplicity of contexts provides for a multiplicity of words and their meanings. It has always been assumed that the word disability means the same thing for everybody in all contexts and that its usage is therefore without limitations. Because words refer to reality, this reality is rightly perceived within its own context and worldview. It is in light of the above assumptions that we have interrogated the word disability and the extent to which it can be seen as disenfranchising if read through the lens of Derrida’s deconstructive hierarchy of binaries as well as the Sesotho linguistic device, *Bitso-lebe-ke seromo*.

The above two optics lend credence to the fact that disability is a disenfranchising category. If read through the lens of the binaries, disability does not occupy the centre. It is a marginal term, which represents the abnormal, the unwanted, the ‘other’ and the imperfect in the physical and moral sense. As long as the word disability carries such a meaning, it permanently denies PWD privileges that all other humans enjoy. Within the Sesotho world view, naming is not a random exercise. It carries immense spiritual power to reflect and indexicalise the behaviours of people. To give a bad name to a person, except for purposes of prevention or survival, is determining someone’s fate on a permanent basis. On that basis, we propose ‘differently abled’ as a designation that better appreciates human diversity while accentuating abilities in every human being.

## Footnotes

1 There is a common practice among many African cultures to dispel a misfortune by giving a weird, unattractive and unpleasant name to the child. A family that suffers a constant loss of children falls under this category.
